# Alligators employ intermetatarsal reconfiguration to modulate plantigrade ground contact

**DOI:** 10.1242/jeb.242240

**Published:** 2021-06-04

**Authors:** Morgan L. Turner, Stephen M. Gatesy

**Affiliations:** 1 Department of Ecology and Evolutionary Biology, Division of Biology and Medicine, Brown University, Providence, RI 02912, USA; 2 Department of Computer Science and Engineering, University of Minnesota, Minneapolis, MN 55455, USA

**Keywords:** Metatarsal, Foot, Alligator, Archosaur, XROMM, Foot posture

## Abstract

Feet must mediate substrate interactions across an animal's entire range of limb poses used in life. Metatarsals, the ‘bones of the sole’, are the dominant pedal skeletal elements for most tetrapods. In plantigrade species that walk on the entirety of their sole, such as living crocodylians, intermetatarsal mobility offers the potential for a continuum of reconfiguration within the foot itself. Alligator hindlimbs are capable of postural extremes from a belly sprawl to a high walk to sharp turns – how does the foot morphology dynamically accommodate these diverse demands? We implemented a hybrid combination of marker-based and markerless X-ray reconstruction of moving morphology (XROMM) to measure 3D metatarsal kinematics in three juvenile American alligators (*Alligator mississippiensis*) across their locomotor and maneuvering repertoire on a motorized treadmill and flat-surfaced arena. We found that alligators adaptively conformed their metatarsals to the ground, maintaining plantigrade contact throughout a spectrum of limb placements with non-planar feet. Deformation of the metatarsus as a whole occurred through variable abduction (twofold range of spread) and differential metatarsal pitching (45 deg arc of skew). Internally, metatarsals also underwent up to 65 deg of long-axis rotation. Such reorientation, which correlated with skew, was constrained by the overlapping arrangement of the obliquely expanded metatarsal bases. Such a proximally overlapping metatarsal morphology is shared by fossil archosaurs and archosaur relatives. In these extinct taxa, we suggest that intermetatarsal mobility likely played a significant role in maintaining ground contact across plantigrade postural extremes.

## Abstract (precis)

**Summary:** We measured 3D metatarsal kinematics in American alligators. Alligator metatarsals conform with the ground across a diversity of high walk and maneuvering postures, providing a context for interpreting the evolutionary history of metatarsals in the fossil record.

## INTRODUCTION

An animal's foot must effectively mediate substrate interactions across the entire range of limb poses used in life. The foot is relatively underexamined in studies of postural and locomotor evolution, which tend to focus on the hip joint (Romer, 1923; Jenkins, 1971; Charig, 1972; Jenkins and Camazine, 1977; Hutchinson and Gatesy, 2000). Even when the distal limb is analyzed functionally, the foot and ankle are often simplified and treated as a ‘black box’ – an anatomically complex set of visually obscured components that are difficult to measure or simulate. Metatarsals, the ‘bones of the sole’, are the dominant skeletal elements of the pes. In plantigrade animals, the heel, metatarsus and phalanges contact the ground during terrestrial locomotion (Hildebrand, 1985; Gebo, 1992; Carrier and Cunningham, 2017). In this foot posture, the entire length of the metatarsus is engaged with the substrate, and individual metatarsals are approximately parallel to the ground surface. Individual metatarsals articulate through a complex network of soft tissues (Schaeffer, 1941; Brinkman, 1980a,b; Cong et al., 1998; Schachner et al., 2011; Suzuki et al., 2011; Hattori and Tsuihiji, 2020), and have been suggested to move independently in some saurians (Brinkman, 1980a; Sullivan, 2007). Such intermetatarsal mobility offers the potential for a continuum of active and passive reconfiguration within the foot itself – a largely unexplored but potentially important contributor to the range of plantigrade limb placements available for an animal to employ.

Extant crocodylians are plantigrade, and have long been recognized to locomote and maneuver using a broad range of hindlimb postures (Cott, 1960; Zug, 1974). From a relatively more erect ‘high walk’, with feet held beneath the body, to a sprawling ‘low walk’, with feet held laterally to the side, this crown group is able to modulate limb pose and foot placement across a postural continuum (Gatesy, 1991; Blob and Biewener, 1999). Because of this diverse repertoire of terrestrial locomotion, living crocodylians have been of interest to paleontologists studying locomotor evolution in Archosauria (Gatesy, 1991; Reilly and Elias, 1998; Hutchinson, 2006; Sullivan, 2015). As such, crocodylian locomotion, particularly the high walk, has been well studied (Schaeffer, 1941; Brinkman, 1980a; Gatesy, 1991; Reilly and Elias, 1998; Blob and Biewener, 1999; Reilly and Blob, 2003; Willey et al., 2004; Reilly et al., 2005; Sullivan, 2007; Baier and Gatesy, 2013; Baier et al., 2018; Tsai et al., 2020). Terrestrial maneuvers (yaws, turns, backing up, striking, etc.) have received substantially less attention. These more disparate behaviors, however, are important to consider in an analysis of locomotor kinematics and functional morphology, as they involve extreme limb and foot poses not typically found in steady forward locomotion.

The crocodylian foot has four dominant, weight-bearing metatarsals, which overlap at their mediolaterally expanded proximal ends. Numerous extinct archosaurs and their relatives also share this overlapping morphology, appearing in basal, croc-line and bird-line taxa, in the early Mesozoic, when these lineages underwent other morphological transitions in the ankle, knee and hip (Tarsitano, 1983; Parrish, 1987; Novas, 1989; Hutchinson, 2006; Nesbitt, 2011; Padian, 2017). Inferred reconstructions of specific taxa and transitions in hindlimb posture remain contentious (Sullivan, 2015). However, an in-depth study of metatarsal motion may elucidate the presence and magnitude of intermetatarsal reconfiguration, as well as the role of overlapping metatarsals in the hindlimb complex.

Measuring individual metatarsal kinematics is challenging. Although studies have been able to infer internal foot kinematics using optical motion capture of external markers in humans (e.g. Simon et al., 2006; Leardini et al., 2007; Jenkyn et al., 2009; McDonald et al., 2016; Welte et al., 2018; Holowka et al., 2021), artifacts of soft tissue movement (Kessler et al., 2019) and an inability to track separate bones hamper resolution. The combination of single-plane X-ray videography with single-plane light videography (Brinkman, 1980a,b; Sullivan, 2007, 2015) led to major advances in our understanding of crocodylian and lizard metatarsal kinematics. However, these studies were limited by low frame rate, an absence of markers, an inability to reconstruct long-axis rotation (LAR), and no treatment of variability in metatarsal motion. Advances in biplanar X-ray videography have enabled high-resolution 3D skeletal movement to be seen and measured inside the foot of avian (Falkingham and Gatesy, 2014; Turner et al., 2020) and human (Kessler et al., 2019; Maharaj et al., 2020) bipeds. The implants necessary for marker-based analysis is challenging in animals with mobile metatarsals, as they are surrounded by many small muscles, nerves and vessels that require careful surgical planning to avoid. Additionally, the biplanar X-ray field of view limits the overall animal size, as the X-rays must often penetrate through the body to capture the distal limb, requiring large markers in the relatively narrow metatarsal shafts to maintain contrast in the X-rays.

This study reports results of *in vivo* 3D metatarsal kinematics of the American alligator, *Alligator mississippiensis* (Daudin 1802), with particular emphasis on plantigrade foot poses across the locomotor and maneuvering repertoire on flat surfaces. The position and orientation of all four weight-bearing metatarsals (metatarsals I–IV) were reconstructed using hybrid X-ray reconstruction of moving morphology (XROMM), a method that combines marker-based XROMM (Brainerd et al., 2010) and scientific rotoscoping (Gatesy et al., 2010). Animated bone models allowed high-resolution measurement of skeletal kinematics using anatomically derived coordinate systems.

The data obtained are used to address three fundamental questions. (1) Does the metatarsus as a whole undergo significant reconfiguration throughout the range of plantigrade postures? (2) If so, what degrees of freedom do metatarsals employ to conform to the ground? (3) What might the dynamic interactions among the metatarsals in alligators reveal about pedal evolution? The crocodylian ability to employ a spectrum of locomotory postures and maneuvers provides an opportunity to look inside the black box and test the role of intermetatarsal mobility in maintaining plantigrade ground contact. Using this new functional perspective, we examine overlapping metatarsal anatomy in the Archosaurian fossil record and infer foot function in extinct members of this posturally diverse clade.

## MATERIALS AND METHODS

### Animals and surgery

Biplanar X-ray data were collected from three female juvenile American alligators, *Alligator mississippiensis* (5.15, 6.25 and 7.09 kg). These animals were initially acquired from the Rockefeller Wildlife Refuge (Grand Chenier, LA, USA) as embryos, captive raised in the alligator colony at California State University, San Bernardino (San Bernardino, CA, USA), then housed in the Brown University Center for Animal Resources and Education. All live animal experiments were conducted in accordance with protocols approved by the Institutional Animal Care and Use Committee of Brown University.

**Fig. 1.**
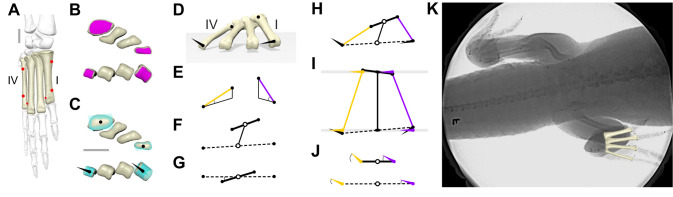
**Quantifying 3D metatarsal motion in *Alligator mississippiensis*.** (A) Dorsal view of the four weight-bearing metatarsals (tan) and six markers (red) used for animation, in the context of other pedal and crural elements (grayscale). (B,C) Selected articular surface patches (magenta; B) and fitted geometric primitives (aqua; C) of proximal (top) and distal (bottom) metatarsals used to calculate centroids (black circles) and cylinder axes (black cones). (D) Anterior perspective view of a hybrid XROMM reconstructed foot pose with proximal and distal centroids and cylinder axes used to calculate metatarsal motion (E–J). Same view of pose used, unless otherwise noted. (E) Pitch of metatarsal I (purple) and IV (gold) long axes measured with respect to ground. (F) Proximal (bold) and distal (dashed) transverse axes, and midpoints (open circles), connected by a middle axis. (G) Skew measured as the 2D rotation of the proximal skew axis with respect to the distal skew axis, as viewed down the middle axis. (H) Centroids, segments and cylinder axes of the metatarsal quadrilateral. (I) Dorsal view of quadrilateral with planes (gray) perpendicular to the middle axis used to project metatarsal I (purple) and IV (gold) cylinder axes. (J) 2D views of proximal (top) and distal (bottom) projection planes, with both transverse axes horizontal to show condylar axis angle measurement. (K) Dorsoventral X-ray frame of high walking on the treadmill, showing animated metatarsals overlain on the right foot. Implanted conical and spherical markers, including many not used in this analysis, are visible. Scale bars: 1 cm.

For marker implantation, animals were induced and maintained on inhaled isoflurane anesthesia within a sterile surgical environment. Radiopaque markers were inserted into metatarsals on both sides of each animal. Implants consisted of either conical markers (0.8 mm diameter and 2–3 mm long) fashioned from carbide steel rods (Kambic et al., 2014) and introduced manually with a pin vise, or 1 mm solid tantalum beads (Baltec, Los Angeles, CA, USA) press-fitted into a hand-drilled 1.0 mm hole. Metatarsals I and IV were each implanted with two markers, spaced as far proximally and distally apart as possible (Fig. 1A) to maximize animation accuracy. Given the anatomical complexity of the soft tissue, we limited our access to the medial (for metatarsal I) and lateral (for metatarsal IV) margins of the pes, navigating between extensor and abductor muscle groups on either side to minimize invasive dissection. A single bead was inserted subcutaneously on the opposite side of the implants near the distal condyle of metatarsals I and IV by inserting a 0.8 mm solid tantalum bead (Baltec) via a hypodermic needle (18 gauge, 3.5 cm long), driven down with the bore of a steel rod plunger. Additional bone and soft-tissue implants were also made in the pelvis and hindlimb for other studies. Skin incisions were closed with 4.0 Vicryl suture (Ethicon Inc.). The alligators ate and behaved normally the day after surgery, and were allowed to recover for at least 1 week before data collection. No signs of locomotor impairment were observed.

### Recording and experimental setup

Walking and maneuvering alligators were recorded at 80 frames s^–^^1^ (1000 μs shutter speed, up to 1800 frames per trial) by two standard light cameras and two X-ray cameras in the W. M. Keck Foundation XROMM Facility at Brown University. This system uses X-ray image chains (Imaging Systems and Service, Painesville, OH, USA) comprised of Varian model G-1086 X-ray tubes (80–90 kV, 200 mA, magnification level 0, 2 ms pulsed beam) suspended by ceiling-mounted telescoping cranes and 16 in diameter mobile-arm mounted Dunlee model TH9447QXH590 image intensifiers (126–140 cm source-to-image distance). Image intensifiers were backed with Phantom v10 high-speed cameras (Vision Research, Wayne, NJ, USA), recording at 1760×1760 pixel resolution and 150 extreme dynamic range. Light video was captured at 1600×1200 resolution using Phantom v9.1 cameras; all cameras were synchronized to within ±4 μs. Images for camera calibration (Knörlein et al., 2016) and undistortion (Brainerd et al., 2010) were recorded before and after each session.

To capture alligator motion diversity, the animals were recorded in two experimental settings: a 35 cm-wide×148 cm-long×48 cm-high acrylic-enclosed motorized treadmill (model DC5, JOG A DOG, Ottawa Lake, MI, USA), and a quadrilateral, acrylic-enclosed arena (wall lengths, 121.9×48.3×31.4×30.5 cm; floor area, 3345.8 cm^2^; height, 38.1 cm) with a 5 cm thick floor of EPS foam (Owens Corning Foamular 150). The tread and foam substrates provided firm, flat, low-slip and level surfaces. The orientations of the two X-ray beams (5 deg and 85 deg from vertical, crossing in a plane nearly transverse to the tread) were chosen specifically to reduce pedal marker occlusion and improve marker contrast from the ground in both recording environments.

### CT scanning, coordinate systems and measurements

After video data collection was completed, animals were euthanized, and the pelvis and hindlimbs were separated from the body. Computed tomography (CT) scans were made with a Nikon X-Tek microCT (Nikon Metrology, Tokyo, Japan) at 120–135 kV, 110–120 μA, 2000×2000 pixel resolution and 0.080–0.127 mm slice thickness. A 0.100 mm Cu filter was placed between the X-ray source and the sample to compensate for beam hardening and reduce artifacts from the metal markers. CT data were reconstructed in Amira 6.0 (Thermo Fisher Scientific) and thresholded surfaces saved as .obj format polygonal models. Models of each element were isolated in Geomagic Wrap 2017 (3D Systems, Morrisville, NC, USA) and cleaned of marker artifacts and internal structure.

Geometric primitives were fitted to polygonal patches (Fig. 1B) manually selected from proximal and distal articular surfaces of the metatarsal models in Geomagic. A cylinder was fitted to the roller-like distal metatarsal condyles and a plane to the proximal metatarsals (Fig. 1C). The centroids of these fitted primitives and the cylinder axes (Fig. 1D) were used to form anatomical coordinate systems (ACSs) for each individual metatarsal, from which more derived measures of bone–bone and bone–ground motion were calculated. Asymmetric ACSs were employed to maintain comparable rotations among right and left feet (following Kambic et al., 2014). Details on coordinate system construction are provided in the Appendix.

The long axes of metatarsals I (purple) and IV (gold) are anatomically static 3D line segments between the proximal and distal primitive centroids of the same metatarsals (Fig. 1E). Metatarsal pitch is the angle each axis makes with its projection onto the ground; we maintain rotational continuity to enable pitch to pass 90 deg. Proximal (solid) and distal (dashed) transverse axes are anatomically dynamic 3D line segments between primitive centroids of metatarsals I and IV (Fig. 1F). Metatarsal spread is calculated as the width of the distal transverse axis divided by the width of the proximal transverse axis, expressed as a percentage.

The midpoints (Fig. 1F,G,H, open circles) of the two transverse segments serve as endpoints of a dynamic middle axis, representing the metatarsus as a whole. This virtual middle axis is used to create two parallel planes (Fig. 1I, gray lines), onto which other previously described 3D axes are projected and measured in 2D. These proximal and distal projection planes include their respective proximal and distal midpoints and are both perpendicular to the middle axis. Skew is calculated as the 2D rotation of the projected proximal transverse axis with respect to the projected distal transverse axis, when viewed distal to proximal along the middle axis (Fig. 1G). The two metatarsal long axes and two transverse axes form the four sides of a dynamic quadrilateral (Fig. 1H). The cylinder axis of each metatarsal condyle (black cone) is likewise projected on the proximal and distal perpendicular planes (Fig. 1I). Projected cylinder axes of metatarsal I (purple cone) and metatarsal IV (gold cone) on these planes permit a simplified 2D view of the quadrangle along the middle axis (Fig. 1J). Metatarsal LARs can then be measured in 2D (Fig. 1J) as the angles of the projected condylar axes with respect to the proximal and distal transverse axes.

### Animation

CT-based metatarsal bone models were animated using a hybrid XROMM method, which combined marker-based XROMM (Brainerd et al., 2010) and scientific rotoscoping (markerless XROMM; Gatesy et al., 2010). As metatarsals were not able to be implanted with a minimum of three markers (a requisite for the marker-based method), they were instead animated using fewer strategically placed markers. These markers serve as 3D-world coordinate anchors that drive anatomically informed animation constraints, serving as a ‘base animation’ that can be further refined through scientific rotoscoping. Here, we followed a three-step process to animate the four dominant alligator metatarsals using hybrid XROMM.

First, unfiltered 3D coordinates of the six metatarsal markers (four implanted, two injected) per foot were extracted in XMALab (Knörlein et al., 2016) and animated in Maya 2020 (Autodesk Inc., San Rafael, CA, USA). These marker coordinates controlled an initial metatarsal I and IV base animation, in which models were positioned based on the implanted markers and given a preliminary orientation based on the injected distal condyle marker. Second, camera calibrations and undistorted X-ray video exported from XMALab were used to create virtual cameras matching the relative positions and orientations of the real-world X-ray sources in Maya. By viewing undistorted video through these virtual X-ray cameras, we refined the initial orientations of the marked metatarsals by aligning the individual bone model to its X-ray shadow in the two views (Gatesy et al., 2010). Finally, the unmarked middle metatarsals (II and III) were given preliminary translations and rotations based on weighted averages of their animated neighbors, followed by rotoscopic refinement.

The marked, outer metatarsals (I and IV) were animated using a two-point rotoscoping method. In each bone, the two implanted markers constrained the base animation such that only one degree of freedom (rotation about an axis between the markers) was left to be controlled. The implanted subcutaneous marker near the distal end of each metatarsal was used to guide the initial rotation animation about this axis. As this injected marker was in mobile soft tissue, this constraint was only used to roughly orient the bone model. Rotation about the implanted marker axis was further refined by rotoscoping. As the implanted markers were on the margin of the bone (medial aspect, metatarsal I; lateral aspect, metatarsal IV), rotation about the axis between these markers made discrepancies in bone-shadow matching apparent. In the case in which an implanted metatarsal marker fell out of the bone into the surrounding tissue and only one rigidly embedded marker remained, a one-point rotoscoping method was used. In this case, the fixed marker was used for model translations and initial rotational animation was guided by the other two markers; all rotations were further refined by rotoscoping.

The unmarked metatarsals II and III were animated using a constrained application of the metatarsal I and IV animation in addition to rotoscoping. Positionally, metatarsal II and III were constrained to the proximal transverse metatarsus axis, such that the proximal end of all four metatarsals were aligned and equally spaced. The orientations of metatarsals I and IV were likewise used to animate preliminary rotations of metatarsal II and III about their proximal centroids, weighted by proximity. Metatarsal I had twice the influence on metatarsal II rotations than metatarsal IV, and metatarsal IV had twice the influence on metatarsal III rotations than metatarsal I. Translations and rotations were further refined by rotoscoping.

### Determination of plantigrade ground contact and data presented in this study

The first and last frames of ground contact of each stance phase were identified from video. For treadmill data, the initial contact of the subsequent stance phase was also identified to constitute a full stride. As light video often captured more of the limbs than the X-ray volume, complete stride frame ranges captured by any camera were used to normalize a partially animated stride. Duty factor was calculated as the fraction of stride duration that a hindlimb was in contact with the ground. As transitions in foot–ground contact were gradual, metatarsal pitch was used to establish a plantigrade threshold. Plantigrady was defined as any stance phase pose with either metatarsal I or IV pitched 15 deg or less. These threshold angles were consistent with transitions in soft-tissue foot contact observed from light and X-ray video.

**Fig. 2.**
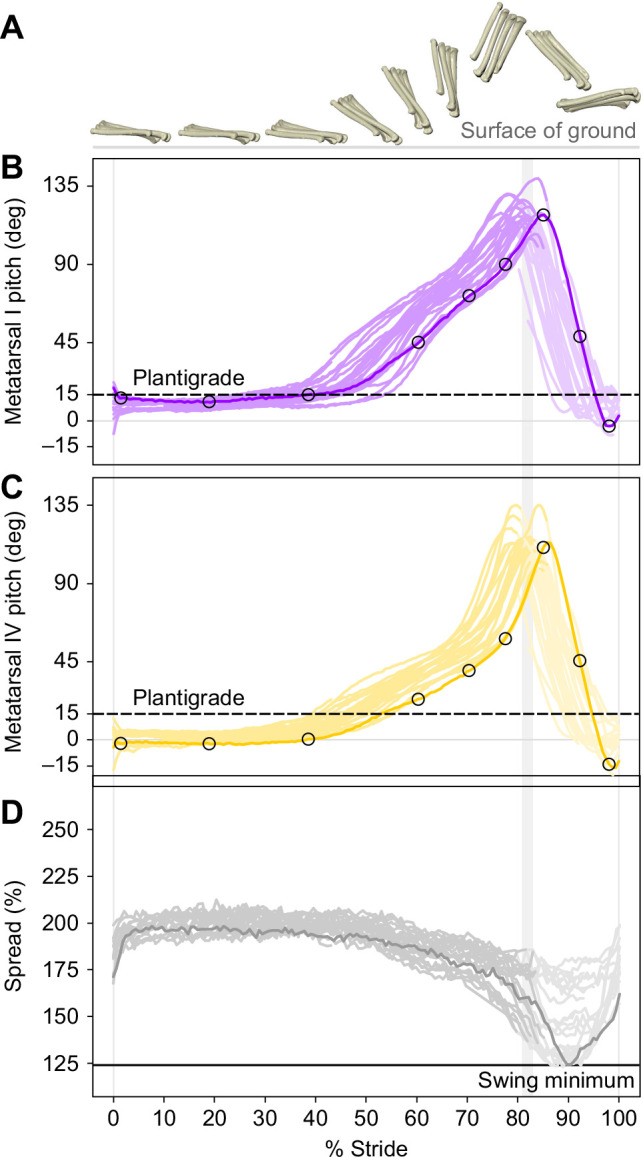
**Metatarsal pitch and spread throughout the high walk stride cycle of *Alligator mississippiensis*.** (A) Lateral view of nine foot poses throughout a representative stride cycle; see Movie 1 for full video. (B,C) Graphs of metatarsals I (B) and IV (C) pitch colored by stance (medium purple/gold) and swing (light purple/gold). (D) Graph of metatarsal spread colored by stance (medium gray) and swing (light gray). Graphs include data from 24 high walk strides of three individuals. The representative stride (dark purple/gold/gray) is circled at the corresponding foot poses in A. A 15 deg pitch threshold (dashed lines) was used for determining plantigrade contact. Vertical bar at mean duty factor (stance/swing transition) drawn at a width of two standard deviations.

**Fig. 3.**
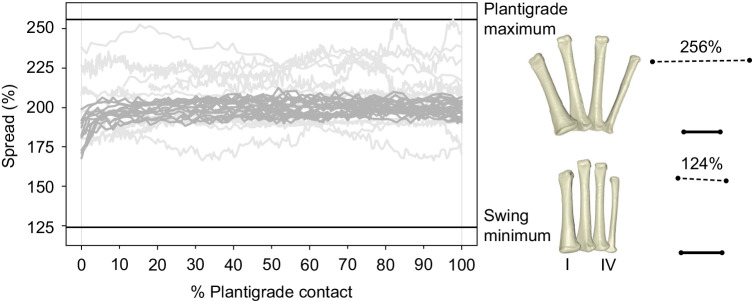
**Metatarsal spread during the plantigrade phase of high walk strides and maneuvers in *Alligator mississippiensis*.** Time normalized to the duration of plantigrade contact. Graph includes data from 24 high walk strides (dark gray) of three individuals and 13 maneuvers (light gray) of two individuals. Maximum and minimum spread (horizontal black lines) match the metatarsal poses (right). Metatarsal poses shown in dorsal view are scaled to metatarsal I length. Diagrams of the same poses show relative length changes of proximal (bold) and distal (dashed) transverse axes.

We analyzed 24 treadmill high walk strides from all three individuals (7, 14, 3), resulting in 3017 frames of data. Given marker placement, pitch and spread measurements were relatively insensitive to metatarsal LAR. Such insensitivity permitted the inclusion of these measurements from additional trials beyond those able to receive rotoscopic refinement, as only small changes in LAR occur when refining the base animation. All 24 high walk strides are presented in Figs 2 and 3. A subset of 13 high walk strides, along with 13 maneuvers, were refined for LAR of the metatarsals, representing the left and right feet of two individuals and resulting in 3924 frames of 6 degree-of-freedom plantigrade data. All figures show right feet; left feet were mirrored and noted. Graphs were created in R (https://www.R-project.org/), and rendered images and videos produced in Maya 2020. Figures were compiled in Adobe Illustrator version 24.3.

## RESULTS

### High walk and maneuvers

Alligators walked on the treadmill for minutes at a time, typically holding the body far off the ground in the well-described high walk ‘semi-erect’ posture (Zug, 1974; Gatesy, 1991; Reilly and Elias, 1998; see Movie 1). However the high walk was not always maintained; animals occasionally would drop hip height to a medium or low walk before either raising back up again or descending to a stop. The feet were placed nearly under the hips pointing anteriorly, and, once in contact with the ground, did not slide. Occasionally, the pes would briefly step on the ipsilateral manus, or a digit would get trapped curled under the pes. High walk strides chosen for analysis excluded significant within-stride hip height variability or toe curl. The 24 strictly high walk strides analyzed had a mean duty factor of 0.82±0.03. Stance-phase duration averaged 1.62±0.31 s, of which about half entailed plantigrade contact (mean, 0.81±0.25 s).

**Fig. 4.**
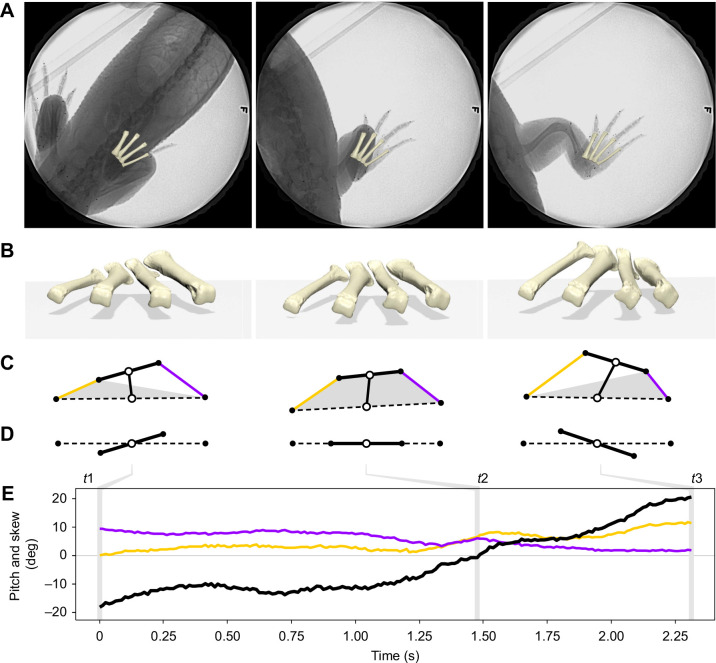
**Metatarsal pitch and skew during a sidestep yaw maneuvering sequence in *Alligator mississippiensis*.** (A) Three dorsoventral X-ray frames representing key timepoints (*t*1, *t*2, *t*3) in sequence; see Movie 2 for full video. (B) Anterior perspective view of metatarsals with shadow projected on ground below. (C) Diagram of metatarsal quadrangle formed by the proximal (bold) and distal (dashed) transverse axes and metatarsal I (purple) and IV (gold) long axes, as defined by the metatarsal centroids (black circles). Transverse axis midpoints (open circles) are connected by the middle axis. Gray shapes represent the base of metatarsal contact. (D) 2D projected views of skewed transverse axes. (E) Graph of skew (black) in the context of metatarsal I (purple) and IV (gold) pitch throughout the maneuver. Vertical lines represent corresponding key timepoints in A–D.

**Fig. 5.**
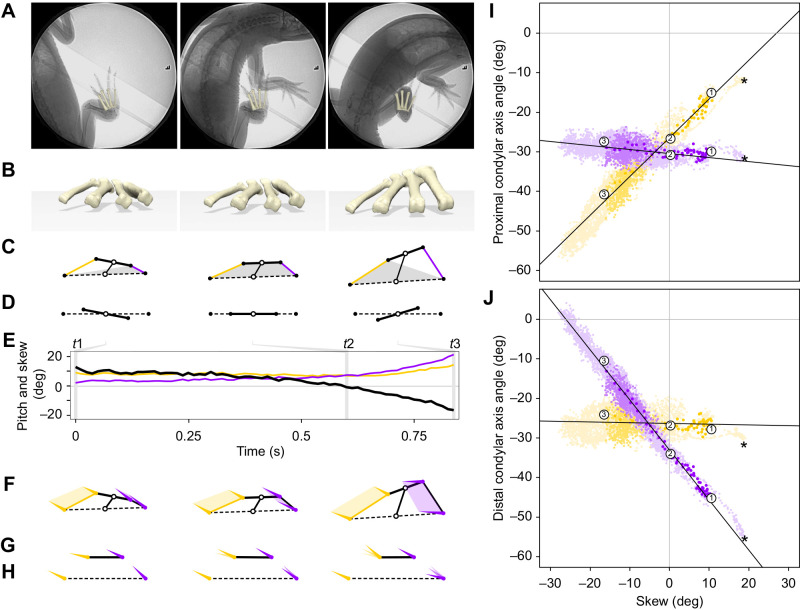
**Projected metatarsal condylar axis angle with respect to skew in *Alligator mississippiensis*.** (A) Three dorsoventral X-ray frames representing key timepoints (*t*1, *t*2, *t*3) in the featured pivot maneuvering sequence; see Movie 3 for full video. (B) Anterior perspective view of metatarsals with shadow projected on ground below. (C) Diagram of metatarsal quadrangle formed by the proximal (bold) and distal (dashed) transverse axes and metatarsal I (purple) and IV (gold) long axes, as defined by the metatarsal centroids (black circles). Transverse axis midpoints (open circles) are connected by the middle axis. Gray shapes represent the base of metatarsal contact. (D) 2D projected views of skewed transverse axes. (E) Graph of skew (black) in the context of metatarsal I (purple) and IV (gold) pitch throughout the maneuver. Vertical lines represent corresponding key timepoints in A–D. (F) Projected condylar axes of poses in (B). (G,H) 2D view of projected cylinder axes with respect to proximal (G) and distal (H) transverse axes. (I,J) Scatterplots with regression lines showing the relationship between skew and proximal (I) or distal (J) projected cylinder axis angles of 3924 poses from 13 maneuvers (light color), and 13 high walks (medium color), representing the left and right feet of two individuals. Featured maneuvers (dark color) with poses at *t*1, *t*2, *t*3 are circled. Asterisks mark pose *t*3 in Fig. 4. In the featured pivot maneuvering sequence, left foot mirrored to appear as right.

Alligators executed a range of maneuvers including tight (pivot) and wide turns while moving forward, sidestep and non-sidestep yaws in place, backing up, low walking with body contact and climbing with forelimbs on the wall. These maneuvers often occurred in combination and were blended rather than discrete. The feet occasionally slid in place against the ground and reoriented. The limbs were never the only point of contact. Often the body, tail and head touched the floor and walls of the enclosure. Plantigrade contact during maneuvers ranged widely; the longest period recorded was 8.9 s (outside foot of a side step yaw, Fig. 4) and shortest was 0.8 s (inside foot of a pivot turn, Fig. 5).

### Metatarsal pitch during the high walk stride cycle

As expected, the metatarsals largely pitched as a single unit during the high walk stride cycle (Fig. 2A; Movie 1); however, the pitch of metatarsal I (Fig. 2B, purple) was almost always higher than that of metatarsal IV (Fig. 2C, gold) during stance. Foot pose at initial contact (0–2% stride) varied from heel first (<0 deg pitch) to toe first (>15 deg pitch) before quickly flattening into full-foot contact. Metatarsal pitch remained under the plantigrade threshold of 15 deg pitch for approximately the first half of stance. During this plantigrade phase, metatarsal IV was often parallel (0 deg pitch) to the ground. Metatarsal I remained above 5 deg pitch, crossing the threshold earlier (mean, 36.5±7.6%) in the stride cycle than metatarsal IV (mean, 45.7±5.5%). As the foot transitioned through digitigrade and unguligrade contact, metatarsal pitch increased, passed vertical (90 deg) and peaked (∼120 deg) at the end of stance.

### Metatarsal spread

Metatarsals spread and collapsed cyclically throughout the high walk stride cycle (Fig. 2D), being most compressed (as low as 124% proximal transverse axis width) when in swing, and most spread (∼200%) when plantigrade. The metatarsals were often highly spread at the start of the stance phase, although spreading was slightly delayed in steps with heel-first contact. Metatarsal spread during the plantigrade phases of the treadmill locomotion pattern provides context for the variation found across maneuvers: the 90 deg spread range during the complete high walk cycles is the same (albeit shifted) as that seen during only the plantigrade phases of maneuvering. Whereas the high walk spread was relatively constant when plantigrade (Fig. 3, dark gray), the metatarsals abducted and adducted much more dynamically in maneuvers (Fig. 3, light gray), reaching a maximum spread of 256%.

### Metatarsal skew when plantigrade

Metatarsal I and metatarsal IV rarely had the same pitch, such that the proximal transverse axis was non-parallel, or ‘skewed’, with respect to the horizontally grounded distal transverse axis. Disparities in metatarsal pitch when plantigrade were far greater during maneuvers than during the high walk (Fig. 2B,C).

Fig. 4 shows pitch and skew data and three sample video frames for a left yaw (see also Movie 2). The sequence begins after the alligator had shifted its body to the right until metatarsal I approached the midline (*t*1). With its right foot still planted, the left limb stepped to the left and turned the body away from the right foot (*t*2). The animal continued moving to the left, extending the right leg laterally as the foot became abducted relative to the body and remained fully engaged with the ground (*t*3). Throughout the sequence, the metatarsals of the right foot remained on the ground distally, but raised and lowered proximally while the pitch of metatarsal I or IV remained under the plantigrade threshold (Fig. 4B). At *t*1, differential pitching of metatarsal I (purple, 9.5 deg) and metatarsal IV (gold, 0.2 deg), led to a −16.7 deg skewing of the transverse axes (Fig. 4C–E). At *t*2, the metatarsals briefly shared the same pitch as the transverse axes passed through a skew of 0 deg. At *t*3, the pitch of metatarsal I was 2.1 deg and IV 11.5 deg, flipping the skew to 18.7 deg.

Thus, alligators maintained plantigrade contact in three different metatarsal configurations. Only rarely were the metatarsals co-planar (0 deg skew). Far more common was for the base of metatarsal I to be elevated above that of metatarsal IV (negative skew, medial raised) or the reverse (positive skew, lateral raised). The diversity of maneuvers analyzed reveal that transitions in skew occurred smoothly from negative to positive (e.g. Fig. 4) and from positive to negative (e.g. Fig. 5) across the total 47 deg range of plantigrade skew measured (−28.0 to 19.1 deg).

### Metatarsal LAR and condylar axis orientation when plantigrade

Skewing of the metatarsus was accompanied by LAR of the individual metatarsals. These relationships are shown by measuring each metatarsal's changing orientation relative to the proximal and distal transverse axes in a pivot to the right (Fig. 5A; Movie 3). The pivot sequence began with the animal having taken a right step backwards, limb extended anterolaterally (*t*1). With its right foot planted, the head and body turned right, and the right manus was placed to the outside of the foot (*t*2). The animal continued to pivot the body about the right foot, which slipped slightly as the foot reached a highly adducted pose relative to the body (*t*3). As in the previous sequence (Fig. 4), the metatarsals pitched and skewed while maintaining plantigrade contact (Fig. 5B–E).

As the foot transitioned from 10.7 to 0.3 to −16.5 deg skew (Fig. 5E), metatarsals were observed to differentially long-axis rotate. Projecting the first and fourth condylar axes onto the two transverse planes revealed distinct modes of metatarsal reorientation. Metatarsal I maintained a relatively consistent orientation (−27.1, −30.9, −27.3 deg at the three sampled times) relative to the proximal transverse axis across the sequence (Fig. 5F,G, purple). By contrast, metatarsal IV underwent significant LAR (−14.2, −26.8, −40.8 deg) with respect to the proximal transverse axis (Fig. 5F,G, gold). The opposite relationships were found when condylar axes were projected on the distal transverse plane (Fig. 5F,H). Metatarsal I reoriented substantially (−44.1, −34.0, −10.5 deg) and IV very little (−26.8, −27.1, −24.3 deg).

These asymmetrical patterns of metatarsal LAR with skew were found among plantigrade data (*N*=3924 poses) from both maneuvers (light color) and high walks (medium color) (Fig. 5I,J, Table 1). The nearly horizontal slopes of the condylar axis angle of metatarsal I (purple, slope −0.10) proximally and metatarsal IV (gold, slope −0.02) distally reveal weak relationships between LAR and skew at these two corners of the metatarsus quadrilateral. On the opposing two corners, the much steeper slope of the condylar axis angle of metatarsal IV (gold, slope 0.98) proximally and metatarsal I (purple, slope −1.27) distally reveal strong inverse relationships between LAR and skew. Maneuvers typically spanned large ranges of skew in a single period of plantigrade contact. The three timepoints from the right-pivot maneuver in Fig. 5I,J (circled) fall near the middle of the pose clouds. Negative skews were more commonly sampled; however, some extreme positively skewed poses were analyzed (e.g. pose *t*3 in Fig. 4, shown as asterisks in Fig. 5I,J). Metatarsal condylar axes were rarely parallel (intersection of purple and gold best-fit line in Fig. 5I,J), which corresponded with an approximate −7 deg skew.

**Table 1.**
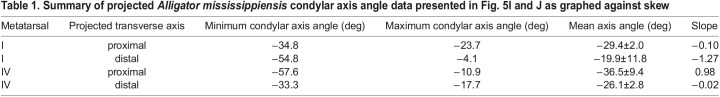
Summary of projected *Alligator mississippiensis* condylar axis angle data presented in Fig. 5I and J as graphed against skew

### Mobility of overlapping proximal metatarsals

**Fig. 6.**
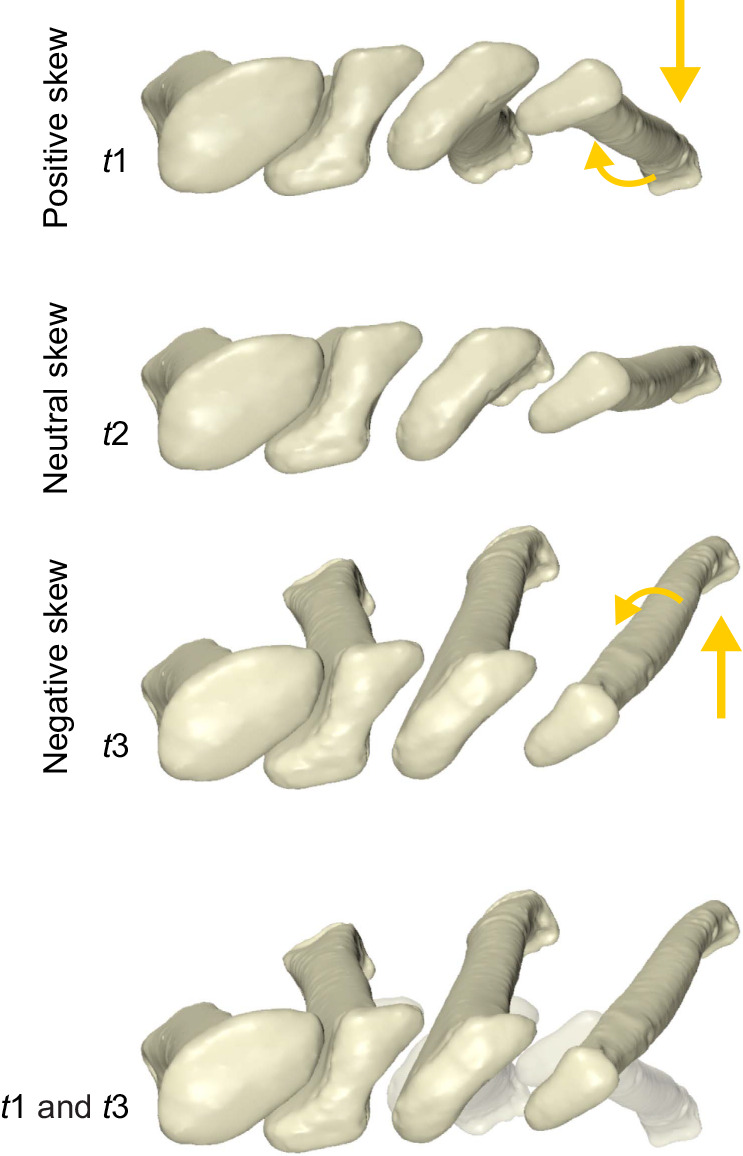
**The changing relationship of overlapping proximal metatarsals with skew in *Alligator mississippiensis*.** Proximal view of key timepoints (*t*1, *t*2, *t*3) in the featured maneuver sequence from Fig. 5, relative to a stabilized metatarsal I. Arrows indicate the direction of coupled long-axis rotation and pitching of metatarsal IV relative to I across the spectrum of skew.

The overlapping proximal metatarsals exhibited a substantial amount of mobility throughout maneuvers involving changes in skew. As metatarsal I maintained a relatively constant relationship with the proximal transverse axis (Fig. 5I, purple), the motion of the other three weight-bearing metatarsals can be visualized relative to a stable first metatarsal (Fig. 6). The same three times sampled from the right pivot featured in Fig. 5 reveal the impact of differential pitch and LAR on proximal metatarsal articulation. Between *t*1 (10.7 deg skew) and *t*3 (−16.5 deg skew), metatarsal IV underwent internal LAR as it dorsiflexed relative to metatarsal I. Metatarsals II and III showed kinematics intermediate between the extremes of metatarsals I and IV. Metatarsal II remained closely apposed to metatarsal I and exhibited little LAR with dorsiflexion. Metatarsal III exhibited moderate degrees of LAR. At negative skews (e.g. *t*1), the proximal bases were most tightly packed as the lateral metatarsals externally rotated and brought the expanded overlapping facets in near contact. At positive skews (e.g. *t*3), internal LAR of metatarsal III and IV increased spacing substantially. Patterns of spacing and proximal reconfiguration seen in the featured maneuver are representative of those observed across the entire spectrum of skew.

## DISCUSSION

This study specifically targeted *in vivo* intermetatarsal mobility of a plantigrade quadruped. Through a hybrid XROMM analysis combining marker-based and markerless XROMM, we were able to visualize, reconstruct and measure the position and orientation of all four dominant, weight-bearing metatarsals in *Alligator*. The ground imposes unique constraints on the metatarsus not typically experienced by more proximal limb segments, and, as such, is an important reference in all analyses performed here. The spectrum of locomotor behaviors sampled affords a quantitative view of previously unseen movements such as spreading and differential pitching, as well as coordination between metatarsal skewing and LAR during plantigrade contact. These kinematic patterns reveal how the long bones of the foot continuously reconfigure to conform with the ground across a diversity of high walk and maneuvering postures. The modulation of the alligator metatarsus under highly varied foot placements and limb postures has potential application in bio-inspired legged robotics, where a machine's ability to span multiple terrain types and locomotor modes (Raibert, 1986; Fukuda et al., 2009) may be aided by novel foot designs beyond the typical shapes [flat, cylindrical and spherical (Ding et al., 2013)] used. Such data on *Alligator* also provide a dynamic context for interpreting the evolutionary history of metatarsals in the fossil record.

### The importance of sampling limb pose diversity in studies of locomotor kinematics and functional morphology

Across all measured variables, metatarsal mobility was greater in maneuvers than in the high walk. This is not surprising, as the cyclical movements of foot and body remained relatively aligned with the direction of the tread. By contrast, foot orientation and placement relative to the body underwent dynamic extremes during maneuvers. In the open arena, the direction of travel often was substantially different from that of the foot, which deviated from the body more variably. A notable difference between these two locomotor modes was the degree of foot slipping on ground. Once planted, the feet did not slip as the body passed over it during the high walk. In maneuvers, particularly yaws and turns, the feet intermittently slid against the ground.

The diversity of foot poses throughout the disparate behaviors presented in this study underscores the importance of sampling non-cyclical behavior in studies of locomotion. As an animal's foot must effectively mediate animal–substrate interactions across its entire repertoire, the morphology and mobility of the metatarsals must function under all possible postural extremes, not just the locomotor mode or gait most commonly used. This disparity in foot function between steady forward locomotion and maneuvering is seen in the *Alligator* data here. Our plantigrade high walk treadmill data sampled only a fraction of the range of spread (Fig. 3) and skew (Fig. 5I,J) found across sampled maneuvers. Subtle patterns of metatarsus deformation and internal reconfiguration can be discerned during the high walk (Fig. 5I,J, medium purple and gold), but are magnified and clarified by the greater range of foot pose extremes (Fig. 5I,J, light purple and gold). XROMM sampling of highly variable locomotor behaviors has revealed previously unseen patterns of hindlimb function (e.g. Kambic et al., 2015; Turner et al., 2020) critical to the interpretation of avian functional morphology. A much larger range of foot placement extremes is found in plantigrade quadrupedal taxa, and thus are critical to incorporate into the study of the locomotor system.

### Intermetatarsal abduction

In plantigrade poses, the metatarsus deformed in two primary dimensions: intermetatarsal abduction (spread) and differential pitching (skew). With skew, metatarsals internally reconfigured through differential LAR. Intermetatarsal abduction was measured as the spreading of the distal metatarsals with respect to a relatively constant proximal metatarsal width. Dynamic spreading occurred throughout the high walk stride cycle, reaching a maximum in the first half of stance and a minimum when in swing (Fig. 2D). Intermetatarsal abduction was relatively constant during plantigrade high walk, and rarely exceeded ∼200% of the proximal width. By contrast, dynamic spreading occurred during plantigrade maneuvers, the maximum (256%) greatly exceeding the high walk plantigrade range (Fig. 3).

The substantially greater spread achieved during maneuvers reveals that the potential limiting factors (bone, cartilage, muscle, ligament, integument and neural recruitment of abductor muscles) are not restrictive to ∼200% spread. Thus, the apparent limit on maximum spread during high walk is instead likely to be due to active forces, such as increased metatarsal adductor muscle activity or decreased load on the metatarsals due to a more cyclical gait. The texture of the tread possibly provided greater frictional resistance to spreading. However, four of the maneuvers recorded here occurred on the treadmill, and were all found to have a maximum spread between 225% and 250%. Future force plate, electromyography and substrate studies designed to explore the interplay of these factors will be useful in elucidating the functional constraints on metatarsal spread during locomotion.

### The plantigrade foot is not flat: metatarsus skewing as a result of differential metatarsal pitch and LAR

Contrary to simplistic depictions of a flat, immobile metatarsus, the four weight-bearing metatarsals have the ability to break from a planar configuration while maintaining plantigrade contact. Such deviations are often referred to as inverted and everted in humans and other taxa (McDonald and Tavener, 1999; Klenerman and Wood, 2006). However, without considering the crus, we cannot quantify inversion and eversion with metatarsals alone, and here focus only on the relationship between the metatarsus and ground. Differences in metatarsal I and IV pitch skewed the metatarsus in both positive (greater metatarsal IV pitch) and negative (greater metatarsal I pitch) directions. Metatarsals of the alligator rarely shared the same pitch, only briefly passing through a ‘flat’ co-planar configuration when transitioning between positive and negative skewed poses (e.g. *t*2 in Fig. 4E, Fig. 5E). Due to such prevalent skewing, the quadrilateral of the metatarsus was most often in a three-point contact formation: both distal metatarsals and either proximal metatarsal I or IV closely engaged with the ground. While the foot was typically visible from only the medial or lateral side in a given stance phase, plantar soft tissue appeared to maintain ground contact on both medial and lateral sides throughout the range of skew found under the plantigrade threshold.

**Fig. 7.**
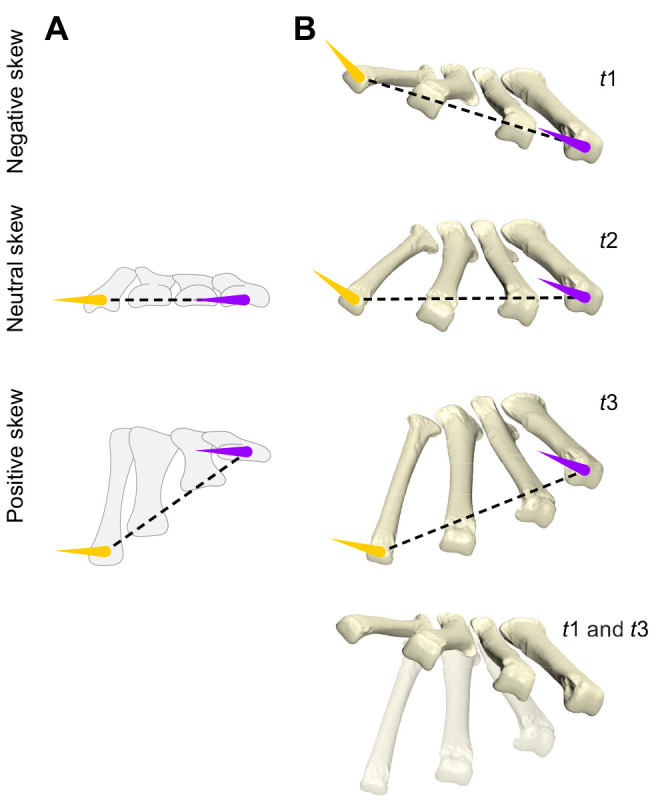
**A comparison of how crocodylian metatarsal skew is achieved in** **Brinkman (1980a)** **and this study.** With stabilized metatarsal I from anterior view, (A) speckled caiman foot poses redrawn from Brinkman (1980a), and (B) alligator foot poses of key timepoints (*t*1, *t*2, *t*3) in the featured maneuver sequence from Fig. 4. Overlain distal (dashed) transverse axes along with projected condyle axes (inferred from drawing in caiman) of metatarsal I (purple) and IV (gold) reveal differences in reconstructed metatarsal long-axis rotation among the two studies.

Brinkman (1980a) identified differential pitching in the spectacled caiman as the source of what he termed ‘metatarsal rotation’ (skew in the present study) (Fig. 7A). Our observations from alligator support the reported caiman pattern of positive skewing (elevated proximal metatarsal IV) in more sprawling poses. However, both Brinkman (1980a) and Schaeffer (1941) interpreted the metatarsals of the spectacled caiman and alligator, respectively, as being entirely flat (thus, 0 deg skew) during the high walk. Whereas the soft tissue of the alligator metatarsus visibly appears flat on the ground, we instead found that, internally, the skeleton was consistently negatively skewed (a more elevated proximal metatarsal I) during treadmill high walk (Fig. 5I,J, medium purple and gold). Given the highly conserved metatarsal morphology among crocodylians, we hypothesize that the metatarsus is negatively skewed during high walk throughout extant members of this clade. Additionally, future work on contextualizing skew with ankle kinematics and limb posture may reveal important insights into how foot contact is achieved across a spectrum of sprawling to erect postures.

Condylar axes, and thus metatarsal LARs, do not maintain a constant relationship during skewing deformations. Contrary to Brinkman's illustrations of parallel condyle axes (Fig. 7A), our alligator data show differential LAR throughout the spectrum of skew (Fig. 7B). Metatarsal I and IV condylar axes were only parallel at an approximate skew of −7 deg, and become increasingly divergent in both extreme positive and negative skew poses. As the condyles were constantly engaged with the ground throughout plantigrade poses, the distal transverse axis can be used as a proxy for horizontal. Given this, the condylar axes were almost always internally rotated (negative LAR) with respect to the ground and the animal was almost always walking on the more medial sides of each metatarsal. Only the metatarsal I condylar axis ever reached parallel with the ground during plantigrade postures, occurring during maneuvers reaching extreme (approximately −25 deg) skew (Fig. 5J). In contrast, metatarsal IV condylar axis was held at a near-constant approximate −25 deg angle with respect to the ground.

Because of the ground constraints on the distal metatarsus, any differential pitching of the metatarsals impacted the relative skew of the proximal transverse axis. Given this geometry, the LAR of individual metatarsals relative to the proximal transverse axis shared an inverse relationship with the distal counterpart. As shown in Fig. 5I, metatarsal I maintained a near-constant (approximate −30 deg) LAR relative to the more proximal transverse axis, and thus the tarsus. By contrast, metatarsal IV rotated over 50 deg LAR for the same range of skew.

This graded LAR of the metatarsals was reflected in the reorientation of their proximal overlapping morphology. More medial metatarsal facets are more horizontally oriented with respect to the ground when at negative skews (e.g. foot beneath the body), and more vertically oriented at positive skews (e.g. during a more sprawling behavior). As the stacked metatarsal arrangement likely precludes underlying lateral metatarsals from pitching above their overlying medial neighbors, vertically reorienting the proximal ends (Fig. 4B, *t*3; Fig. 5B, *t*1) would appear to take advantage of the axis of freedom they do have – medial metatarsals dorsiflex and lateral metatarsals plantarflex. In the extreme negative skews, the expanded metatarsal heads stack horizontally (Fig. 4B, *t*1; Fig. 5B, *t*3) relative to the ground and may offer stability within the foot when beneath the body.

The combination of differential metatarsal pitch and differential metatarsal LAR permits extremes in foot–ground contact to be accommodated within the metatarsus. Metatarsal I LAR is relatively static to the proximal metatarsus transverse axis (Fig. 5I, purple) and metatarsal IV LAR relatively static to the distal transverse axis (Fig. 5J, gold). As such, metatarsal I primarily moves with the tarsus and rolls against the ground, whereas metatarsal IV registers with the ground and rolls against the ankle. The strong correlation between differential LAR and skewing suggests that these movements are mechanically linked with more proximal elements within the foot. Extremes in spacing among the proximal metatarsals support involvement of other anatomical structures (articular cartilage, joint capsules, ligaments, muscles, tendons), as the bones are not simply sliding past one another.

### Overlapping metatarsals in extinct reptiles

**Fig. 8.**
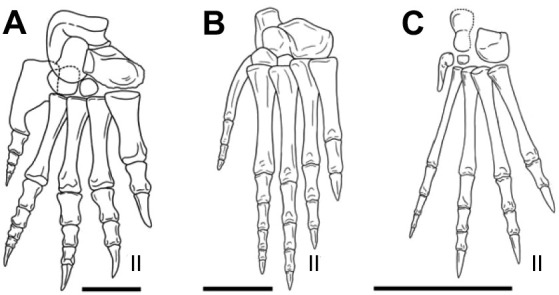
**Diagrammatic images of right pedal skeletons of three croc-line archosaurs.** (A) *Stagonolepis robertsoni* (redrawn from Walker, 1961), mirrored to show as right*.* (B) *Ticinosuchus ferox* (redrawn from Krebs, 1965). (C) *Protosuchus richardsoni* (redrawn from Colbert and Mook, 1951). Digit II indicated on each foot. Scale bars: 0.5 cm. Reproduced from Farlow et al. (2014) with permission from Wiley under the terms of the CC-BY 4.0 license.

The presence of overlapping metatarsals is among the first major shifts from a basal amniote ‘mosaic’ ankle (Schaeffer, 1941; Thulborn, 1980; Sumida, 1989; Laurin and Reisz, 1995) into an ‘integrated’ diapsid pes (Gauthier et al., 1988). Several significant changes in foot and ankle structure evolved throughout diapsid clades [e.g. hooked fifth metatarsal (Lee, 1997; Sullivan, 2010; Borsuk-Bialynicka, 2018), reduction of distal tarsals (Joyce et al., 2013), ‘rotary’ proximal tarsal joint (Sereno and Arcucci, 1990)]. Croc-line archosaurs retain this primitive metatarsal condition (Fig. 8) and all share a similar ankle structure (Parrish, 1987; Farlow et al., 2014). Our findings of metatarsal function in *Alligator* are most appropriately considered within this clade and provide dynamic context for interpreting fossil morphology.

With such potential for continuous internal reconfiguration, the morphology of the overlapping metatarsals in croc-line archosaurs do not directly correspond with any one skeletal arrangement with respect to the ground, but rather speak to the overall mobility of the metatarsus as a whole. Although this metatarsal mobility is likely to be an advantage for the semi-aquatic *Alligator*, the patterns of metatarsal movement under the constraint of ground contact provides a valuable reference for terrestrial biomechanical reconstructions of croc-line archosaurs. Given the mobility and kinematic relationships of the metatarsus revealed in *Alligator*, we propose several functional constraints when reconstructing or mounting fossil croc-line archosaur hindlimbs. (1) Extinct plantigrade taxa need not have held their metatarsal condyles parallel to the ground. Metatarsals were likely almost always internally rotated when engaged with relatively flat terrain. (2) Proximal metatarsal heads need not be maximally congruent – open gaps were likely present among the lateral metatarsals depending on the metatarsus skew. (3) Metatarsal alignment can go beyond planar, even if raising the base of metatarsal I above lateral metatarsals appears non-intuitive. (4) Metatarsal spread should be greater in stance than swing, but can expand further during maneuvers. We propose spreading the distal metatarsals 200% that of the proximal width as a starting hypothesis for plantigrade poses during high walk of all croc-line archosaurs.

Adducted, fused, appressed or compact metatarsal configurations are found to have convergently evolved in several erect/cursorial tetrapod lineages. Such feet with at least the proximal half of metatarsals II–IV contacting each other appear in pterosaurs, some crocodylomorphs, as well as most ornithischian, sauropodomorph and theropod taxa (character 382 in Nesbitt, 2011). The likely reduction of metatarsal mobility associated with such intermetatarsal contact suggests a reduction or complete loss of metatarsus-based ground conformation within these groups, and thus taxa with adducted metatarsals were not likely to be plantigrade. Indeed, pterosaurian (Padian, 1983) and dinosaurian (Holtz, 1995; Jannel et al., 2019) lineages, along with few croc-line taxa [e.g. *Poposaurus gracilis* (Farlow et al., 2014; Schachner et al., 2020)], are suggested to be digitigrade and rely on the toes to maximize ground contact. As many taxa throughout Archosauria exhibit varying degrees of adduction or fusion along the length of the metatarsus, we suggest that this character is likely to be an indicator of the degree of metatarsus conformation possible within the pes. Continued work on the mechanical relationship of intermetatarsal adduction/abduction, foot posture and limb posture may provide a novel perspective on the evolution of erect posture in Archosauria, as foot and limb posture are suggested to be correlated (Coombs, 1978; Hutchinson, 2006).

Although metatarsal morphology and kinematics are one piece of the locomotor system whole, the results here show that intermetatarsal mobility likely plays a significant role in maintaining ground contact in plantigrade Archosaur species with complex rotary ankles and greater postural extremes. Considering the variability and unevenness of natural terrain, the ability for the foot to deform is likely an advantage for conforming to and pushing off from a variety of surfaces. It is likely that this ability to deform shares a significant relationship with foot placement and limb posture, and may be a key functional trait contributing to the locomotor diversity recorded in the Archosaurian fossil record.

## Supplementary Material

Supplementary information

## Figures and Tables

**Fig. A1.**
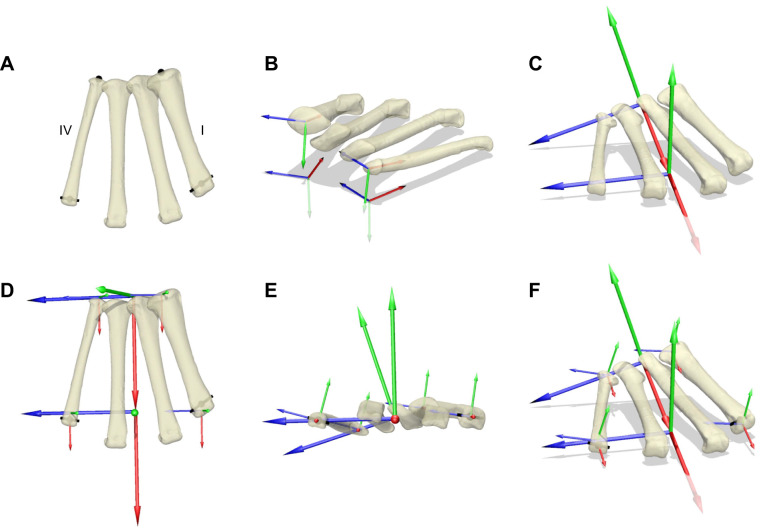
**Metatarsal coordinate system construction on a single reconstructed foot pose of an alligator right foot.** (A) Proximal and distal metatarsal centroids (black circles) and condylar axes (black bars) of metatarsals I and IV used to construct all coordinate systems in this study. (B) Pairs of MTpitchACS and MTgroundACS axes in posterior perspective view used to calculate pitch of metatarsals I and IV. (C) Pair of proximalProjectedTransverseACS and distalProjectedTransverseACS in anterior perspective view used to calculate skew. (D–F) Projected condylar axes shown in relationship to proximal and distal transverse axes and condylar axes in dorsal (D), distal (E) and anterior (F) perspective views. Shadows projected onto ground plane in B, C and F. Rotations about the red *x*-axis, green *y*-axis, and blue *z*-axis follow right-hand rule conventions for positive rotations.

## APPENDIX

### Details on coordinate system construction

Construction details of dynamically calculated anatomical coordinate systems (ACSs) (Fig. A1) in Autodesk Maya 2020. Language specific to this software is used herein. All coordinate systems were constructed using the centroids of the fitted geometric primitives and cylinder axes (Fig. A1A), as outlined in the Materials and Methods (Fig. 1B–D). All coordinate systems were designed to isolate and measure specific movements in 2D. Asymmetries in right and left systems were employed to maintain comparable rotations among all feet (following Kambic et al., 2014). Right-foot coordinate system construction is detailed here; differences in left-foot coordinate system construction are noted in each respective section.

**Pitch** (Fig. A1B): The translate_X and translate_Z component of the metatarsal plane centroid formed the origin of the MTgroundACS. Translate_Y was locked at the height of the ground surface (Y positive ventrally). MTgroundACS was locked in rotate_X and rotate_Z, so that it maintained the same relationship to the ground plane. MTgroundACS_X was aim constrained at the centroid of the metatarsal cylinder, only rotating about MTgroundACS_Y. The centroid of the metatarsal plane formed the origin of the MTpitchACS. MTpitchACS_X was aim constrained at the centroid of the metatarsal cylinder, with a Y up vector to MTgroundACS (Y positive down). The *z*-axes of both MTpitchACS and MTgroundACS remained parallel to the ground surface (resulting in Z positive medially) and were perpendicular to the long axis of the metatarsal, permitting a 2D dynamic measurement of pitch, regardless of foot orientation in space. Identical construction was followed for the left foot, resulting in X positive distally, Y positive ventrally and Z positive laterally.

**Skew** (Fig. A1C): The midpoint of the metatarsal I and IV metatarsal plane centroids formed the origin of proximalProjectedTransverseACS. proximalProjectedTransverseACS_X was aim constrained at the midpoint of the metatarsal I and IV metatarsal cylinder centroids (X positive distally), with a Z up vector to the metatarsal IV metatarsal plane centroid (Z positive laterally). The midpoint of the metatarsal I and IV metatarsal cylinder centroids formed the origin of distalProjectedTransverseACS. distalProjectedTransverseACS_X was aim constrained at the midpoint of the metatarsal I and IV metatarsal plane centroids (X positive distally), with a Z up vector to metatarsal IV cylinder centroid (Z positive laterally). The *x*-axes of both proximalProjectedTransverseACS and distalProjectedTransverseACS represent the midline of the metatarsus (see also Fig. A1D,E), and permit a dynamic 2D measurement of skew, regardless of metatarsal configuration. Construction for left feet differed only in sign of both aim constraints and up vectors, such that the resulting axes were X positive proximally, Y positive dorsally and Z positive laterally.

**Projected condylar axes** (Fig. A1D–F): Four projected condylar axes (a pair of proximal and distal for each metatarsal) were constructed in two steps. First, condylar axis aims were created, then projected condylar axes were constructed.

Condylar axis aim objects were created for metatarsals I and IV. MT1condylarAim was point constrained to the metatarsal I cylinder centroid and orient constrained to the metatarsal I cylinder axis, such that MT1condylarAim_Z was in line with the cylinder axis. Both point and orient constraints were deleted. MT1condylarAim was parent constrained to the cylinder axis, then translated along the *z*-axis several centimeters off to the lateral side of the foot. The same was repeated for metatarsal IV.

A pair of proximal and distal projected condylar ACSs was created for each metatarsal. We detail the construction of metatarsal I distal projected condylar ACS here. MT1distalProjectedCondylarACS was point and orient constrained to distalProjectedTransverseACS. Constraints were then deleted. MT1distalProjectedCondylarACS was parented under distalProjectedTransverseACS. MT1distalProjectedCondylarACS translate_X, translate_Y and rotate_Y, rotate_Z were locked. MT1distalProjectedCondylarACS translate_Z was point constrained to metatarsal I cylinder centroid. MT1distalProjectedCondylarACS_Z was aim constrained at MT1condylarAim (Z positive laterally), constraining axes to only rotating about X, no up vector. All four projected condylar axes were constructed in the same manner, using respective centroids and metatarsal condylar axis aim object. The *y*- and *z*-axes of all projected condylar axes remained within the planes of the projected transverse axes. Rotation about the *x*-axis permitted a dynamic 2D measurement of condyle axis angle, regardless of metatarsal configuration. Identical construction was followed for the left foot, resulting in axes X positive proximally, Y positive dorsally and Z positive laterally.
