# Supplementary figures and images for: Alligators employ intermetatarsal reconfiguration to modulate plantigrade ground contact
Source: J Exp Biol. 2021 Jun 4;224(11):jeb242240. doi: 10.1242/jeb.242240 (PMC8214830; doi:10.1242/jeb.242240)

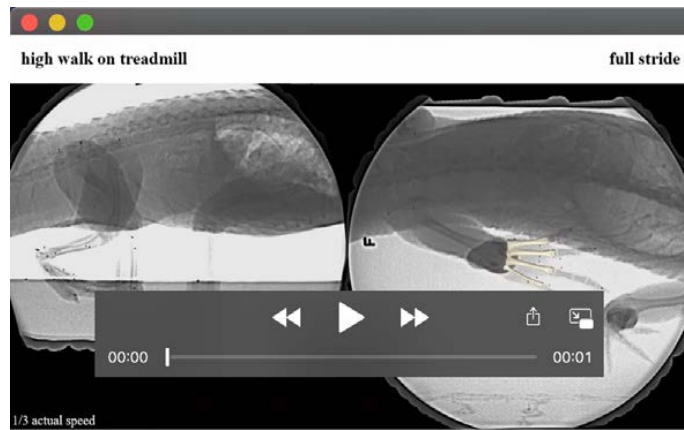

**Movie 1.** High walk on treadmill.

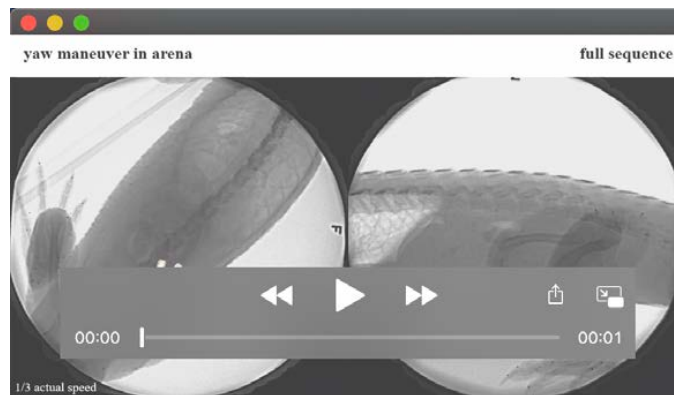

**Movie 2.** Yaw maneuver in arena.

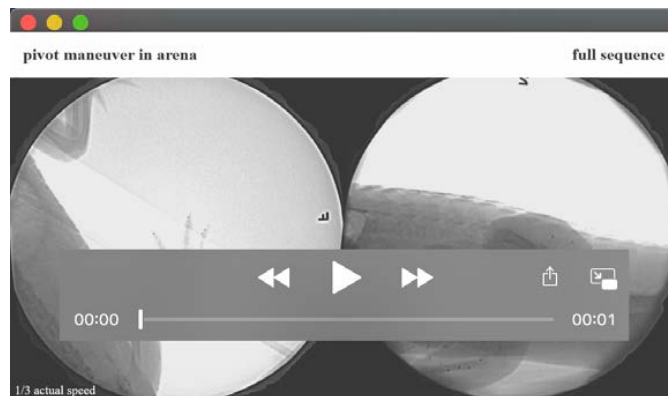

**Movie 3.** Pivot maneuver in arena.

Supplement: Supplementary information [file jexbio-224-242240-s1.pdf]
